# The Association between Obesity-Risk Genes and Gestational Weight Gain Is Modified by Dietary Intake in African American Women

**DOI:** 10.1155/2018/5080492

**Published:** 2018-03-01

**Authors:** Ying Meng, Susan W. Groth, Dongmei Li

**Affiliations:** ^1^Clinical and Translational Science Institute, University of Rochester, Rochester, NY, USA; ^2^School of Nursing, University of Rochester, Rochester, NY, USA

## Abstract

Obesity-risk genes have been associated with dietary intake, appetite regulation, and gestational weight gain (GWG). The purpose of this study was to examine whether dietary intake including total energy intake and macronutrients modify or mediate the association between obesity-risk genes and GWG. An observational study was conducted with 85 African American pregnant women. Sociodemographic, medical, and lifestyle factors and dietary recalls were collected during pregnancy. Seven obesity-risk genetic variants were genotyped. Regression analyses with bootstrapping methods were used to examine the moderation and mediation effects of dietary intake. The mean GWG was 14.2 kg, and 55.3% of the women gained above the Institute of Medicine GWG guidelines. A nominally significant association was found between rs17782313 (close to *MC4R*) and percentage of energy intake from fat (*P*=0.043). A variant downstream of *KCTD15* (rs11084753) was nominally significantly related to GWG (*P*=0.023). There was a significant interaction between the *KCTD15* polymorphism and dietary fat intake (*P*=0.048). Women with the *AG* genotype gained more weight during pregnancy with more dietary fat consumption. In conclusion, our results indicate that dietary macronutrients, especially fat intake, may modify the effect of the *KCTD15* gene on GWG. Improved knowledge of gene-diet interactions can facilitate the development of personalized interventions.

## 1. Introduction

High gestational weight gain (GWG) has been linked to adverse pregnancy and birth outcomes, such as preeclampsia, cesarean delivery, and high birthweight [1]. High GWG also has been associated with long-term maternal weight retention and offspring obesity [2, 3]. According to the US Institute of Medicine (IOM) 2009 guidelines [4], approximately 48% of women gain weight excessively during pregnancy [5]. African American women have a higher rate of weight retention and obesity after delivery compared to Caucasian women [6]. Overall, more than 38% of US women are obese, and among them, African American women have the highest rate (57%) of obesity [7].

Obesity is a consequence of an imbalance between energy intake and energy expenditure [8]. To date, the mechanism of how and why an imbalance occurs has not been fully understood. The regulation of energy homeostasis is complex and involves multiple factors, such as neural and endocrine systems related to food intake, environments linked to food availability, and sedentary lifestyles associated with energy expenditure [8, 9].

Genes involved in the body systems that regulate energy homeostasis potentially contribute to the development of obesity. Genome-wide association studies have been used to identify obesity-susceptible variants in nonpregnant populations [10, 11]. Most of the identified variants are within or close to genes that are highly expressed in the hypothalamus, a region that controls dietary intake and regulates energy balance [10]. For example, several variants in or near the fat mass and obesity-associated (*FTO*) gene, the melanocortin 4 receptor gene (*MC4R*), the SH2B adaptor protein 1 gene (*SH2B1*), the potassium channel tetramerization domain containing 15 gene (*KCTD15*), the neuronal growth regulator 1 gene (*NEGR1*), and the brain-derived neurotrophic factor gene (*BDNF*) have been associated with satiety, poor eating behaviors, and dietary intake (e.g., high energy and high fat intake) [12–15].

In addition to genes having a direct involvement in body energy regulation, several studies have found that genetic effects on body weight can be modified by environmental factors, such as dietary macronutrients. A higher body mass index (BMI) was reported in the *FTO* (rs9939609) *AA* carriers who consumed a high-fat diet compared to the *TT* carriers [16]. An interaction between the *A* allele and protein intake was also observed [15]. Similarly, a greater BMI was shown in the apolipoprotein A2 (*APOA2*, rs5082) *CC* carriers who consumed a diet high in saturated fat compared to *TT* or *TC* carriers [17]. Protein intake has been found to modify the effect of the *MC4R* gene on obesity-related traits, such as appetite and food craving [18].

Genes that have been associated with obesity in the general population are likely to have an influence on GWG because pregnancy is characterized by rapid weight gain within a relatively short period of time and has been linked to long-term maternal and offspring obesity [2, 3]. Several studies have examined the effect of obesity-risk genes, such as *FTO*, *MC4R*, and the transmembrane protein 18 (*TMEM18*) on GWG [19–22]. However, the impact of individual genes on GWG remains uncertain. A possible explanation is that the effect of a single gene on GWG is minimal. Another explanation is that the impact of certain genes could be modified by environmental factors similar to what are seen in nonpregnant populations. The varied responses of genotypes to environmental factors during pregnancy may mask the overall effect of certain genes on GWG.

Therefore, the purpose of this study was to investigate the impact of obesity-risk genes on weight gain and dietary intake during pregnancy. In addition, the mediation and modification effects of dietary intake on GWG were examined, which to our knowledge has not been assessed previously in pregnant women.

## 2. Methods and Materials

### 2.1. Study Population

The study was approved by the university institutional review board for human subject research. It was a prospective cohort study in which African American pregnant women were followed longitudinally throughout pregnancy and early postpartum [19]. Ninety-seven women were recruited from two urban obstetrical clinics from 2008 to 2011. Participants were followed from less than 20 weeks of gestation until 6 months postpartum. Women were included if they were 18 years of age or older, enrolled in Medicaid and/or the Special Supplemental Nutrition Program for Women, Infants, and Children (WIC), and had singleton pregnancy, and their prepregnancy body mass index (BMI) fell between 18.5 kg/m^2^ and 40.0 kg/m^2^. Women were excluded if they had medical or psychological conditions that prevented them from signing the consent form or had medical conditions that could potentially influence GWG (e.g., diabetes or hypertension before pregnancy). For the current study, women who delivered prior to 35 weeks of gestation, which would influence total GWG, and/or who had gestational diabetes, which could affect dietary patterns during pregnancy, were excluded. Women who had missing dietary data during pregnancy were also excluded. The final sample consisted of 85 participants. The sample size for individual genes varied from 72 to 85 due to different genotype call rates.

### 2.2. Dietary Data

Dietary intake was collected during the following three periods of pregnancy: 16–22 weeks, 24–29 weeks, and 32–37 weeks. For each dietary assessment, 24-hour dietary recalls were obtained using the Nutrition Data System for Research software version 2009 (University of Minnesota Nutrition Coordinating Center, Minneapolis, MN), a Windows-based nutrition analysis program. A trained technician interviewed participants either face to face or via telephone. A multiple-pass method was utilized to enhance the complete recall of food intake [23]. Each 24-hour recall took approximately 30–45 minutes. Total energy intake and percentage of calories from fat, carbohydrate, and protein were calculated based on the dietary recalls. In order to adjust for daily variation of dietary intake, estimated usual intake during pregnancy was calculated using the protocol designed by the National Cancer Institute [24].

### 2.3. Anthropometric and Sociodemographic Measurements

Maternal weights were retrieved from prenatal medical records. Gestational weight gain was determined by subtracting the weight assessed during the initial prenatal visit from the final weight recorded before delivery. If the initial prenatal visit was after 14 weeks of gestation, the weight was adjusted by deducting the average weekly weight gain during the 2nd trimester specifically for African American women in order to minimize an underestimation of GWG [4]. GWG was also classified into three categories based on prepregnancy BMI in accordance with the 2009 IOM guidelines: low weight gain, appropriate weight gain, and high weight gain. The IOM guidelines vary depending on prepregnancy BMI: women who have a normal prepregnancy BMI (18.5–24.9 kg/m^2^) are advised to gain between 25 and 35 pounds; women who are overweight before pregnancy are advised to gain between 15 and 25 pounds; and women who are obese before pregnancy are advised to gain between 11 and 20 pounds [4].

Information about maternal age, height, education, marital status, and health insurance was collected at enrollment through questionnaires and abstracted from medical records. Prepregnancy BMI was calculated based on maternal weight at their initial prenatal visit and height (kg/m^2^). Educational attainment was the highest grade completed at the time of enrollment. Marital status was categorized into two groups: married/have a partner and separated/no partner. Health insurance was reported as public or private.

### 2.4. Other Variables

Gestational information about parity, estimated gestational age at delivery, and gestational diabetes were retrieved from medical records. Parity was grouped into three categories: no previous pregnancy, one prior pregnancy, and more than one prior pregnancy. Lifestyle factors (i.e., smoking and illicit drug use) during pregnancy were also retrieved from medical records. Smoking and illicit drug use were both dichotomized into two groups (yes/no). Physical activity was assessed by the Pregnancy Physical Activity Questionnaire (PPAQ) [25] during early pregnancy (16–22 weeks) and was used in the current study. The PPAQ is a validated questionnaire which includes items that focus on time spent on 32 activities and provides an estimate of average weekly energy expenditure (MET-hours/week) calculated by multiplying the reported time by the intensity of each activity.

### 2.5. Genotyping

Saliva samples were obtained from the participants at enrollment using Oragene DNA self-collection sampling kits (DNA Genotek Corporation, Ottawa, Canada) by trained study coordinators. Genotyping of seven obesity-risk single-nucleotide polymorphisms (SNPs) were performed at the University Genomics Research Center [20, 26]. Additional information about the seven SNPs and sample sizes is provided in Supplementary Table 1. The pairwise linkages among the seven SNPs were less than 0.1, which indicated no strong linkage disequilibrium among the SNPs.

### 2.6. Statistical Analysis

Descriptive statistics were calculated to summarize the sample characteristics. To obtain normally distributed variables, log transformation was performed on total energy intake, and inverse transformation was performed on percentage of energy intake from protein. General linear models were used to assess the genetic effect on GWG and dietary intake. Parity and illicit drug use were related to GWG (*P* < 0.1) and therefore were controlled in the analyses of GWG. Maternal age, prepregnancy BMI, smoking, and marital status were related to total energy intake (*P* < 0.1) and were adjusted in the analyses of dietary intake. Because the obesity-related SNPs might be associated with prepregnancy BMI, the relationship between these SNPs and prepregnancy BMI was examined using general linear models. The *P* values ranged from 0.075 to 0.975. Prepregnancy BMI was included in the analyses of dietary intake since there was no strong association between this variable and the SNPs. Estimated marginal means of the genotypes were compared using the Bonferroni correction and 1000 bootstrapping resamples. A *P* value less than 0.05 was considered as nominally significant. A *P* value less than 0.007 was considered as significant after adjusting for multiple testing with seven SNPs.

The mediation and moderation models were performed using the PROCESS macro in SPSS [27]. Model 1 in Hayes's PROCESS was used to assess the genetic moderation effect. Model 4 was used to test the mediation effects of dietary factors between genotypes and GWG. Maternal age, prepregnancy BMI, smoking, marital status, parity, and illicit drug use were controlled in both models. The 95% confidence intervals (CIs) were estimated with 5000 bootstrapping resamples. All statistical analyses were conducted using SPSS version 22.0.

## 3. Results

### 3.1. Characteristics of the Participants

The characteristics of the 85 African American women are presented in Table 1. The GWG ranged from losing 3.6 kg to gaining 39.5 kg, and the mean GWG was 14.2 kg. More than 55% of the women gained weight above the 2009 IOM recommendations. The average daily energy intake during pregnancy was ∼2386 kcal/day. Maternal age ranged from 18 to 35 years. The mean prepregnancy BMI was 28.4 kg/m^2^, and 68.2% of the women were overweight or obese (BMI ≥ 25 kg/m^2^). The majority of women had at least one prior pregnancy (55.3%), was married or had a partner (77.6%), had a high school or higher education (55.3%), and had public insurance (90.6%).

### 3.2. Associations between Genotype and Dietary Intake

The association between the polymorphism rs17782313, close to the *MC4R* gene, and the percentage of energy intake from fat was significant at the nominal level but not after adjusting for multiple testing (*P*=0.043). The *TC* carriers had a relatively lower fat intake than the *TT* carriers (Bonferroni *P*=0.038 and bootstrap *P*=0.022) when controlling for maternal age, prepregnancy BMI, smoking, and marital status (Table 2).

### 3.3. Mediations between Genotype, Dietary Intake, and Gestational Weight Gain

A nominally significant association was observed between the polymorphism rs11084753, located around 15.5 kilobase pairs downstream of the *KCTD15* gene, and GWG (*P*=0.023). The *AG* carriers gained more weight during pregnancy (mean: 16.9 kg; bootstrap 95% CI: 14.5 kg to 19.4 kg) compared to the *GG* carriers (mean: 12.3 kg; bootstrap 95% CI: 10.2 kg to 14.5 kg; Bonferroni *P*=0.057 and bootstrap *P*=0.018) and the *AA* carriers (mean: 10.9 kg; bootstrap 95% CI: 7.3 kg to 15.0 kg; Bonferroni *P*=0.09 and bootstrap *P*=0.026).

The mediation effect of dietary intake on the association between the genotype of rs11084753 and GWG was not significant. The mediation effects (Path *a* × *b*) of percentage of energy intake from fat and carbohydrate were significant on the associations between GWG and the SNPs, rs5443 and rs17782313. But neither the total effects (Path *c*) nor the direct effect (Path *c*′) of these SNPs on GWG was conclusive (Supplementary Table 2).

### 3.4. Interactions between Dietary Intake, Genotype, and Gestational Weight Gain

**Figure 1:**
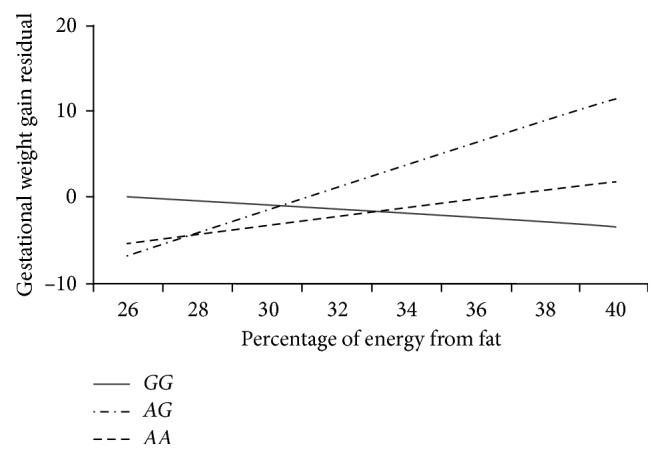
Interactions between rs11084753 close to *KCTD15* and percentage of energy intake from fat on gestational weight gain. Gestational weight gain residual was obtained by adjusting for maternal age, smoking, marital status, prepregnancy BMI, parity, and illicit drug use. Regression lines represent the expected relationship between dietary fat intake and gestational weight gain for individual genotypes.

The effect of gene-diet interactions on GWG is presented in Table 3. The polymorphism rs11084753, close to the *KCTD15* gene, was nominally significantly interacted with percentage of energy intake from fat (*P*=0.048). The moderation effect of this polymorphism on the relationship between fat intake and GWG was found in the *AG* carriers (*P*=0.016). Women who possess this genotype (regression coefficient: 1.29; 95% CI: 0.60, 1.97; *P*=0.001) gained more weight during pregnancy, when they consumed more fat (Figure 1). The *AG* genotype was also significantly interacted with percentage of energy intake from carbohydrate (*P*=0.042). This interaction between the *AG* genotype and carbohydrate intake was potentially due to the negative correlation between carbohydrate intake and fat intake (correlation coefficient: −0.782; *P* < 0.001).

## 4. Discussion

In the current study, the effect of the gene-diet interactions on weight gain during pregnancy was assessed. The SNP rs17782313, located close to the *MC4R* gene, was nominally significantly associated with the percentage of energy intake from fat during pregnancy. The mediation effect of dietary intake during pregnancy on GWG was not conclusive. The SNP rs11084753, located downstream of the *KCTD15* gene, had a nominally significant relationship with GWG and interacted with the percentage of energy intake from fat and carbohydrate. The *AG* carriers gained more weight during pregnancy, and their GWG was higher with greater amounts of dietary fat intake.

The SNP rs17782313, mapped downstream of the *MC4R* gene, has been associated with body weight and obesity [28]. *MC4R* is expressed in the hypothalamus and plays a role in the regulation of eating behavior and appetite [29]. Several studies have examined the relationship between this SNP and dietary fat intake in nonpregnant populations. A significant positive association between the SNP and the absolute total fat intake was found in two previous studies [30, 31] but not in two other studies [13, 32]. In our study of African American pregnant women, the SNP was nominally significantly related to percentage of energy intake from fat. The *C* allele carriers had relatively lower fat intake during pregnancy. However, the effect of this SNP was not conclusive after corrections for multiple testing. Therefore, further investigation is warranted to determine whether this SNP close to the *MC4R* gene has an effect on dietary fat intake during pregnancy.

The SNP rs11084753, mapped downstream of the *KCTD15* gene, was previously linked to BMI with the *G* allele increasing the risk of obesity in nonpregnant individuals [11]. In this study, a nominally significant association was observed between the SNP and GWG. The *AG* carriers gained more weight during pregnancy than the *AA* carriers. Stuebe et al. detected a similar trend of higher GWG in the *G* allele carriers, although the effect was not significant [20]. We also found novel significant interactions between the SNP and macronutrient intake of fat and carbohydrate during pregnancy. In our participants, fat intake was correlated with carbohydrate intake, which could potentially explain why this SNP interacted with both fat and carbohydrate intake. Specifically, the results demonstrated that the *AG* carriers gained more weight during pregnancy when they consumed a diet high in fat. Despite a lack of extensive research on the *KCTD15* gene, studies with animal models have indicated a relationship between this gene and obesity-related behaviors [33]. Williams et al. found that *KCTD15* and another obesity-related gene (*AP-2β*) colocalized in regions of the mouse brain known to control feeding behaviors, including the hypothalamus [33]. Also, these two genes directly interacted in a mouse hypothalamus-derived cell line. In the *Drosophila* model, *KCTD15* is involved in the regulation of the number and size of meals. In the chicken model, feeding, fasting, and a high-fat diet significantly altered the expression level of the *KCTD15* gene in the hypothalamus and adipose tissue, suggesting a possible association between *KCTD15* and dietary fat intake [34].

There are some limitations to this study that should be considered. First, all participants self-reported as African Americans and ancestry markers were not examined to delineate the participants in a more precise manner. Second, the 24-hour dietary recall was self-reported. However, the Nutrition Data System for Research is a validated tool for collection of dietary information [35], and a technician specially trained to use the system collected the dietary data. A multiple-pass method was utilized to enhance food intake recall [23]. In addition, a statistical method designed by the National Cancer Institute was applied to calculate estimated usual dietary intake to adjust for daily variation of dietary intake [24]. Although multiple methods were employed to strengthen the accuracy of the dietary data collection, an imprecision of food intake may have occurred and could attenuate the association between the SNPs and dietary intake. Last, the sample size was relatively small. Yet, as all participants were self-reported as African Americans, the homogeneity of the sample may have enhanced the power to identify genetic associations. Nevertheless, the nonsignificant results should be interpreted as inconclusive, as the sample size was not adequate to detect a small effect size. Future replications with larger samples and different race/ethnicity groups are recommended.

In conclusion, the current study demonstrated that the SNP linked to the *KCTD15* gene potentially had an impact on gestational weight gain, and this effect might be modified by dietary fat intake. In addition, the SNP close to the *MC4R* gene was possibly associated with dietary fat intake. This study highlighted potential genetic predisposition to weight gain during pregnancy and possible modification by environmental factors, such as dietary intake. From a public health perspective, the findings from this study and future nutrigenetic studies could contribute to the development of personalized risk identification and individualized dietary recommendations for pregnant women.

## Figures and Tables

**Table 1:** Characteristics of the sample (*N* = 85).

Characteristic	Value
*Outcomes*	
Gestational weight gain (kg), mean (SD)	14.2 (±7.4)
Gestational weight gain-IOM^a^, number (%)	
Appropriate weight gain	23 (27.1)
Low weight gain	15 (17.6)
High weight gain	47 (55.3)
*Dietary intake*	
Total energy intake (kcal/day), mean (SD)	2385.8 (±488.0)
Percentage of calories from fat, mean (SD)	32.7 (±2.7)
Percentage of calories from carbohydrate, mean (SD)	55.0 (±3.8)
Percentage of calories from protein, mean (SD)	13.6 (±1.6)
*Demographic factors*	
Age, mean (SD)	23.4 (±4.7)
Prepregnancy BMI (kg/m^2^), mean (SD)	28.4 (±5.5)
Education (grade), median (range)	12 (3–16)
Marital status (married/have a partner), number (%)	66 (77.6)
Health insurance (public), number (%)	77 (90.6)
*Medical and lifestyle factors*	
Parity (nulliparous), number (%)	38 (44.7)
Gestation weeks, median (range)	39.4 (35.4–41.1)
Smoking, number (%)	21 (25.0)
Illicit drug use, number (%)	22 (26.5)
Physical activity (MET-hours/week^b^), mean (SD)	286.6 (±117.1)

^a^Calculated based on the 2009 IOM guidelines; ^b^MET means metabolic equivalent.

**Table 2:** The effect of the genetic variant close to the *MC4R* gene on dietary fat intake.

Genotype		Fat% (mean)	95% confidence interval^a^		*P* ^b^
rs17782313 (*MC4R*)	*TT*	33.5	32.7	34.2	0.043
	*TC*	32.0	31.1	32.9	
	*CC*	32.7	31.6	34.1	

^a^95% confidence interval estimated using 1000 bootstrap resampling; ^b^general linear model adjusted for maternal age, prepregnancy BMI, smoking, and marital status.

**Table 3:** The effect of gene-diet interactions on gestational weight gain.

		Gestational weight gain^a^				
		*β*	95% confidence interval		*P*	*P*_interaction_
Percentage of energy intake from fat		−0.34	−1.38	0.69	0.51	
*KCTD15* (rs11084753)	*AG*	3.44	0.30	6.57	0.03	
	*AA*	−0.02	−4.56	4.51	0.99	
	*GG*	Reference				
Percentage of energy intake from fat × *KCTD15*	*AG*	1.80	0.35	3.25	0.02	0.048
	*AA*	0.96	−0.73	2.65	0.26	
	*GG*	Reference				
Percentage of energy intake from carbohydrate		−0.01	−0.57	0.54	0.96	
*KCTD15* (rs11084753)	*AG*	3.56	0.40	6.71	0.03	
	*AA*	−0.59	−5.70	4.52	0.82	
	*GG*	Reference				
Percentage of energy intake from carbohydrate × *KCTD15*	*AG*	−0.80	−1.57	−0.03	0.04	0.10
	*AA*	−0.03	−2.11	2.06	0.98	
	*GG*	Reference				

^a^Process model 1 adjusted for maternal age, smoking, marital status, prepregnancy BMI, parity, and illicit drug use.

## Supplementary Materials

Supplementary Materials Supplementary Table 1: SNPs genotyped in the current study. Supplementary Table 2: regression analyses testing the mediation effect of dietary intake on the association between obesity-related genes and gestational weight gain.
