# Supplementary material for: The Association between Obesity-Risk Genes and Gestational Weight Gain Is Modified by Dietary Intake in African American Women
Source: J Nutr Metab. 2018 Mar 1;2018:5080492. doi: 10.1155/2018/5080492 (PMC5852892; doi:10.1155/2018/5080492)
Supplement: Supplementary Materials — Supplementary Table 1: SNPs genotyped in the current study. Supplementary Table 2: regression analyses testing the mediation effect of dietary intake on the association between obesity-related genes and gestational weight gain. [file 5080492.f1.pdf]

Supplemental Table 1. SNPs genotyped in the current study.

**SNP**	**Nearest Gene**	**Genotype**	**Sample Ssize**
rs5443	*GNB3*	*TT*	41
	CDCA3	*CT*	36
		CC	8
rs9939609	*FTO*	*TT*	15
		AT	31
		AA	26
rs17782313	*LOC342784*	*TT*	43
	ENSG00000267620	*CT*	32
	MC4R	*CC*	4
rs11084753	*KCTD15*	*GG*	33
		AG	39
		AA	12
rs7498665	*SH2B1*	*AA*	50
		AG	30
		GG	3
rs2568958	*LOC105378797*	*AA*	19
	NEGR1	*AG*	41
		GG	22
rs10938397	*THAP12P9*	*AA*	41
	GNPDA2	*AG*	31
		GG	2

Supplemental Table 2. Regression analyses testing the mediati~~ng~~on effect of dietary intake on the association between obesity-related genes and gestational weight gain

		Path *c*			Path *c'* and *b*			Path *a*			Path *a* x *b*			
		B	SE	p	B	SE	p	B	SE	p	B	SE	LLCI	ULCI
GNB3	CT vs TT	1.07	1.83	0.56	2.27	1.87	0.23	-1.36	0.75	0.07	-1.20	0.70	-2.97	-0.09
Fat%					0.88	0.31	0.006							
MC4R	CT vs TT	0.06	1.97	0.98	1.48	1.93	0.45	-1.40	0.73	0.06	-1.42	0.92	-3.84	-0.11
Fat%					1.02	0.32	0.002							
MC4R	CT vs TT	0.06	1.97	0.98	1.17	1.97	0.55	1.93	1.06	0.07	-1.11	0.75	-3.30	-0.08
Carb%					-0.57	0.19	0.004							

Process model 4 adjusted for prepregnancy BMI, maternal age, smoking, marital status, illicit~~egal~~ drug use, and parity.

Formatted: Font: 12 pt
